# Knowledge and Attitude of Eastern Province Family Physicians Toward Common Orthopedic Surgery Cases

**DOI:** 10.7759/cureus.101756

**Published:** 2026-01-17

**Authors:** Ammar Alomran, Manar Alossaif, Hadi Alhamal, Abdulaziz Alwusaibie, Sarah Alhaddad, Dalal Albaiji, Abdullah H Alnasser, Abdulaziz A Alfayez

**Affiliations:** 1 Department of Orthopedic Surgery, King Fahad University Hospital, Dammam, SAU; 2 College of Medicine and Surgery, Imam Abdulrahman Bin Faisal University, Dammam, SAU; 3 Orthopedic Surgery, Dammam Medical Complex, Dammam, SAU; 4 College of Medicine, Imam Abdulrahman Bin Faisal University, Dammam, SAU

**Keywords:** diagnostic accuracy, orthopedic knowledge, orthopedic referrals, primary care physicians, referral guidelines

## Abstract

Background

Orthopedic outpatient departments often experience long waiting lists, even though many referred cases do not require specialist evaluation. Referral guidelines were introduced to address unnecessary referrals and have been continuously updated; however, knowledge gaps persist, particularly in pediatric orthopedic cases. This study aims to evaluate the accuracy of diagnoses and referrals made by Primary Health Care Physicians (PCPs) compared to established referral guidelines in the Eastern Province of Saudi Arabia.

Methodology

This cross-sectional study was conducted among family physicians in the Eastern Province using a self-administered questionnaire with clinical scenarios of common orthopedic conditions and multiple-choice options to assess diagnoses, appropriate management, and referral decisions.

Results

Our results showed that PCPs demonstrated better diagnostic accuracy for knee osteoarthritis (OA) and frozen shoulder in comparison to Developmental Hip Dysplasia (DDH) and scoliosis. Most PCPs advised bed rest for acute low back pain, which is against the recommendations. Physicians with greater clinical experience showed more accurate diagnostic skills.

Conclusion

Primary care physicians generally have an acceptable understanding of common orthopedic conditions, such as knee osteoarthritis and frozen shoulder, and have a clear preference for conservative management approaches. However, notable knowledge gaps persist, particularly in DDH. Furthermore, there were gaps in their knowledge of correct management approaches for back pain and scoliosis. Additionally, a considerable portion of participants remain unfamiliar with existing referral guidelines, highlighting the need for enhanced training in orthopedic assessment and appropriate referral practices.

## Introduction

Orthopedic outpatient departments frequently encounter overwhelming waiting lists, the majority of which stem from unnecessary referrals that do not require specialist intervention. This issue persists despite efforts to implement referral guidelines aimed at improving the quality of referrals. The American Academy of Pediatrics (AAP) initially introduced referral guidelines for pediatric surgical specialists in 2002 to address concerns about excessive referrals to pediatric orthopedic clinics [1,2]. However, Reeder et al. (2004) [3] found that 42% of referrals to pediatric orthopedic clinics did not adhere to these guidelines, highlighting a significant gap in diagnostic skills and understanding of musculoskeletal (MSK) conditions among primary care providers [4].

This lack of progress persisted, as demonstrated by Hsu et al.'s 2012 study, which reported no significant reduction in the rate of inappropriate referrals [5]. In response, the AAP updated its referral guidelines in 2014 and launched an annual musculoskeletal (MSK) Boot Camp in 2016 to better equip primary care physicians with the necessary skills to manage MSK conditions [6,7]. Nevertheless, a 2022 study revealed that only 50% of referrals adhered to the updated guidelines, indicating that knowledge gaps and diagnostic challenges remain prevalent [8].

Upon reviewing the literature, two studies have highlighted the ongoing issue of inappropriate referrals, including Balazs et al., who reported that 48% of pediatric orthopedic referrals were deemed inappropriate, with many conditions manageable at the primary care level [8]. Similarly, Nashi et al. found that 74.5% of pediatric orthopedic referrals could have been managed by primary care physicians, underscoring the need for improved education and clearer referral criteria [6].

Innovative approaches are being explored to address these challenges. National Health Service (NHS) England’s 2023 guidance on referral optimization in MSK care emphasizes the value of utilizing specialist advice services to ensure patients are directed to the most appropriate healthcare provider at the right time, thereby reducing unnecessary referrals to secondary care. This model promotes better integration between primary, community, and secondary care by leveraging MSK expertise already present within primary care settings, ultimately improving case management and patient outcomes [9].

This study aims to evaluate the knowledge and attitudes of family physicians in the Eastern Province of Saudi Arabia toward common orthopedic cases, with a particular focus on the appropriateness of their referrals to orthopedic surgery departments. By assessing these factors, we aim to identify knowledge gaps that contribute to unnecessary referrals and provide recommendations to enhance referral practices in accordance with established guidelines.

## Materials and methods

Study subjects

Primary health care physicians (PCPs) in the Eastern Province of Saudi Arabia constituted the study population in this cross-sectional research, which aimed to assess the knowledge and attitudes of family medicine residents, fellows, specialists, and consultants toward common orthopedic surgery cases.

Sampling and sample size

Initially, the expected proportion (p) was set at 0.54 based on findings from the pilot phase of the study, in which 63 out of 116 primary care physicians demonstrated fully correct diagnostic recognition across all three orthopedic case vignettes. This proportion was selected as it directly reflects the anticipated level of adequate diagnostic knowledge in the target population under similar clinical scenarios. The minimum required sample size was calculated using the single population proportion formula, taking the proportion of PCPs with fully correct diagnosis in all three orthopedic case vignettes from the pilot data (p=0.54), a 95% confidence level (Z = 1.96), and an absolute precision of 10% (d = 0.10). This yielded an initial sample size of 96 participants. After inflating by 10% to compensate for potential non-response, the final minimum required sample size was 107 PCPs. The achieved sample of 116 PCPs therefore exceeded the minimum required, ensuring adequate precision for the primary outcome. Furthermore, the formula used to calculate the sample size is n=[Z^2^_α/2 _p(1 - p)]/d^2^, where Z_α/2_=1.96 for 95% confidence, p=0.54 (expected proportion with good diagnostic knowledge), and d=0.10 (acceptable absolute precision of ±10%).

Materials

Data were collected over an eight-month period using a self-administered questionnaire. The questionnaire consisted primarily of case scenarios representing common orthopedic conditions frequently encountered in primary care settings. It was developed by the authors following a comprehensive literature review and subsequently revised and validated by three expert orthopedic consultants.

Variables

Respondents’ demographic data included gender, age, job role, years of experience, and place of employment. Additionally, two items were used to self-evaluate participants’ orthopedic knowledge: the first utilized a 5-point scale (1 = very poor, 2 = poor, 3 = fair, 4 = good, 5 = very good), and the second was a yes/no question assessing awareness of existing orthopedic referral criteria. Five clinical case scenarios of common orthopedic conditions were included to evaluate diagnostic knowledge and referral attitudes using multiple-choice questions. Furthermore, one additional item used to assess the general attitude toward referring orthopedic causes using a multiple-response format in which participants can select more than one option

Procedure of data collection

The questionnaire was developed in an online format and distributed through digital platforms, including WhatsApp and Telegram, over an eight-month period. After data collection, responses were exported into Microsoft Excel, where the dataset was filtered and organized for statistical analysis.

Data analysis

After data collection, responses were coded and entered into a structured datasheet, and statistical analysis was performed independently by a bio-statistician not involved in the study team to minimize analytical bias. The dataset was analyzed using the SPSS software, version 26.0 (IBM Corp., Armonk, NY). Categorical variables, including gender, qualification, residency year, employment center, and referral criteria awareness, were presented as numbers and percentages. Continuous variables derived from composite Likert-based section scores were summarized using mean ± standard deviation and observed ranges. The readiness domain consisted of two questions (score range: 2-10), attitude domain contained four questions (score range: 4-20), confidence domain included six questions (score range: 6-30), responsibility domain analyzed four questions (score range: 4-20), barriers section comprised four questions (score range: 4-30), and the final barriers-to-care domain contained 4 questions (score range: 4-30). Mean section scores were computed for each domain, and total domain scores were later dichotomized into negative or positive attitude categories based on distribution-derived thresholds, with higher scores indicating more positive physician readiness or management attitude. Group differences in knowledge and attitude/management scores across physician characteristics were evaluated using Mann-Whitney U and Kruskal-Wallis H tests. A p<0.05 was considered statistically significant.

## Results

The sociodemographic and professional profile of Primary care physicians (PCPs) is given in Table 1. A total of 116 primary care physicians participated in the study, of whom 63 (54.3%) were female, and 53 (45.7%) were male. The sample was predominantly composed of residents, accounting for 86 (74.1%) participants, followed by specialists at 18 (15.5%) and consultants at 12 (10.3%). Among resident physicians, 47 (54.7%) were in R1, 27 (31.4%) in R2, and 12 (14.0%) in R3. Fellowship training among consultants was reported by four (33.3%), while eight (66.7%) did not hold a fellowship. Most participants had between one and five years of clinical experience after internship in the primary healthcare field as general physicians, residents, specialists, or consultants, representing 84 (72.4%), whereas 22 (19.0%) had 5-10 years and 10 (8.6%) had more than 10 years of experience. Employment or training was primarily within Ministry of Health facilities, reported by 79 (68.1%) physicians, followed by the Imam Abdulrahman Bin Faisal University (IAU) Family and Community Centers at 22 (19.0%), Armed Forces Hospital-Dhahran at seven (6.0%), Royal Commission Hospital-Jubail at four (3.4%), and both Johns Hopkins Aramco Healthcare (JHAH) and King Fahd Military Medical Complex (KFMMC), Dhahran at two (1.7%) each. With respect to self-rated orthopedic knowledge, 57 (49.1%) physicians rated their knowledge as fair, 27 (23.3%) as poor, 13 (11.2%) as very poor, 18 (15.5%) as good, and one (0.9%) as very good. Awareness of established referral criteria for orthopedic-related conditions was reported by 66 (56.9%) physicians, while 50 (43.1%) indicated no such awareness.

**Table 1:** Sociodemographic and professional profile of primary care physicians (PCPs)

		N	%
Gender	Female	63	54.3
	Male	53	45.7
Qualification/ position	Consultant	12	10.3
	Specialist	18	15.5
	Resident	86	74.1
If resident, year of residency (n=86)	R1	47	54.7
	R2	27	31.4
	R3	12	14.0
If consultant (any fellowship) n=12	No	8	66.7
	Yes	4	33.3
Years of Experience (After Internship)	1-5 Years	84	72.4
	5-10 Years	22	19.0
	More Than 10 Years	10	8.6
Training/Employment Center	Ministry of health	79	68.1
	IAU Family and community center	22	19.0
	Armed Forces Hospital - Dhahran	7	6.0
	JHAH	2	1.7
	KFMMC - Dhahran	2	1.7
	Royal Commission Hospital- Jubail	4	3.4
How much do you rate your knowledge in orthopedic-related conditions on a scale (1-5)	1 (Very Poor)	13	11.2
	2 (Poor)	27	23.3
	3 (Fair)	57	49.1
	4 (Good)	18	15.5
	5 (Very Good)	1	.9
Are you aware if there are any referral criteria related to any orthopedic-related condition	No	50	43.1
	Yes	66	56.9

Primary care physicians demonstrated high diagnostic accuracy for degenerative knee disease, correctly identifying osteoarthritis in 108 (93.1%) cases, and the majority also selected appropriate initial management, with 83 (71.6%) favoring combined lifestyle modification, Non-Steroidal Anti-Inflammatory Drugs (NSAIDs), and physiotherapy. In the infant hip vignette, 76 (65.5%) correctly identified developmental dysplasia of the hip, while 71 (61.2%) chose appropriate action with ultrasound and orthopedic referral, although 31 (26.7%) selected delayed follow-up. In the scoliosis scenario with a Cobb’s angle greater than 48 degrees, only 47 (40.5%) appropriately selected orthopedic referrals, whereas reassurance and stretching were incorrectly chosen by 59 (50.9%). For the frozen shoulder vignette, most physicians demonstrated correct diagnostic knowledge, with 95 (81.9%) identifying adhesive capsulitis, and 81 (69.8%) selected optimal conservative management with physiotherapy and NSAIDs. In contrast, management of acute nonspecific low back pain showed substantial variability, with 74 (63.8%) incorrectly recommending bed rest, while only 38 (32.8%) selected appropriate delayed referral based on symptom severity or functional limitation (Table 2).

**Table 2:** PCP knowledge of diagnosis and attitude toward appropriate management/referral PCP: Primary care physician, NSAIDs: Non-Steroidal Anti-Inflammatory Drugs, SCFE: Slipped Capital Femoral Epiphysis, US: Ultrasound, PTH: Parathyroid hormone, OA: Osteoarthritis.

Case Scenario	Domain	Response Option	N (%)	Interpretation
A 64-year-old male presented to the clinic complaining of pain in his knees that started one year ago. Upon further questioning, the pain is limited to the anterior and medial sides bilaterally. It is also difficult to bend his knees when he is sitting on the floor, and climbing the stairs makes the pain worse. On local examination, there were no scars, skin rashes or erythema. However, there was varus deformity bilaterally and a limited range of motion with crepitus on passive movement.	Knowledge	A. Rheumatoid arthritis	1 (0.9%)	Incorrect diagnosis
		B. Septic knee	2 (1.7%)	Incorrect diagnosis
		C. Osteoarthritis*	108 (93.1%)	Correct knowledge
		D. Patellofemoral pain syndrome	5 (4.3%)	Incorrect diagnosis
	Attitude	A. Refer per the patient’s request	4 (3.4%)	Not evidence-based
		B. Lifestyle modification + NSAIDs	25 (21.6%)	Partially appropriate
		C. Refer to physiotherapy	3 (2.6%)	Partially appropriate
		D. Both B & C*	83 (71.6%)	Best attitude
A 2-month-old baby girl came to the vaccination clinic, while you were evaluating her you noticed leg length discrepancy, you did a hip examination and you noticed limitations in hip abduction.	Knowledge	A. Physiological bowing	31 (26.7%)	Incorrect knowledge
		B. SCFE	7 (6.1%)	Incorrect knowledge
		C. Developmental Dysplasia of Hip*	76 (65.5%)	Correct knowledge
		D. Proximal femoral focal deficiency	2 (1.7%)	Incorrect knowledge
	Attitude	A. Double diaper advice	14 (12.1%)	Not recommended
		B. Do US + refer to Orthopedic surgery*	71 (61.2%)	Correct attitude
		C. Follow-up 2 months + re-evaluate	31 (26.7%)	Acceptable but delayed
		D. Reassurance only	0 (0%)	Incorrect action
An 18-year-old girl presented to your clinic after her mother noticed a shoulder imbalance. Upon further questioning, there were no respiratory symptoms, no walking issues, and no back pain. On examination, there were no skin dimples, no rash, no scars, or hairy patches. You performed Adam’s forward bending test and there was noticeable asymmetry. You ordered X-Ray and the image confirms the presence of a bend to the right and Cobb’s angle >48 degrees.	Attitude	A. Reassure + stretching	59 (50.9%)	Inappropriate
		B. Refer to Orthopedic surgery*	47 (40.5%)	Appropriate attitude
		C. Order Vit D, Ca, PO₄, PTH	4 (3.4%)	Unnecessary
		D. Encourage bracing	6 (5.2%)	Not sufficient for angle >48°
A 50-year-old woman with diabetes presents with a 2-month history of insidious onset of right shoulder pain. She denies a history of shoulder trauma. She has no history of neck pain, arm/hand weakness, numbness or paresthesia. The pain is felt at extremes of range of motion, and the patient has difficulty sleeping on the affected side. She has noticed increasing difficulty with activities of daily living, including brushing her hair, as well as putting on or taking off her clothes. Her examination shows a marked decrease in both active and passive range of motion of the right shoulder.	Knowledge	A. Rotator cuff tear	16 (13.8%)	Incorrect knowledge
		B. Frozen shoulder*	95 (81.9%)	Correct knowledge
		C. Shoulder impingement	4 (3.4%)	Incorrect knowledge
		D. Glenohumeral OA	1 (0.9%)	Incorrect knowledge
	Attitude	A. Physiotherapy alone	15 (12.9%)	Partially appropriate
		B. Ortho referral on 1st request	7 (6.1%)	Not evidence-based
		C. NSAIDs alone	13 (11.2%)	Partially appropriate
		D. Both A & C*	81 (69.8%)	Best attitude
A 26-year-old female presents with acute low back pain. She says it started a week ago after she lifted a sofa when helping a friend move. The patient's medical history is otherwise negative. The patient says the pain is limited to the lower back. The physical examination is normal, including the neurologic examination.	Attitude	A. Bed rest	74 (63.8%)	Incorrect attitude
		B. Twisting/bending only	4 (3.4%)	Harmful advice
		C. Immediate ortho referral	0 (0%)	Partially appropriate
		D. Ortho referral 2nd visit if severe or limits function*	38 (32.8%)	Most appropriate attitude

An additional item was used to assess the general attitude toward referring orthopedic causes using a multiple-response format in which participants can select more than one option (Table 3). The general reasons for referral to orthopedic clinics among primary care physicians showed that most physicians (n = 113) referred patients when they were not responding to initial management, whereas referral based on patient request was the least common reason (n = 30). 

**Table 3:** Reasons of orthopedic referrals from PCPs

Question	Domain	Response options	Number of participants who chose the following option
Generally, in your practice, what would make you refer a case to orthopedic surgery? (you can choose multiple options)	Attitude	Patients’ request	30
		Unsure of the diagnosis	105
		The need for further investigations (laboratory investigations and imaging)	102
		Not responding to initial management	113
		No available physiotherapists in the center	65

The proportion of primary care physicians who correctly recognized diagnoses across the three orthopedic case vignettes varied across physician characteristics (Table 4). Consultants and specialists demonstrated higher proportions of fully correct diagnoses, with nine (75.0%) consultants and 15 (83.3%) specialists achieving correct identification in all three cases, compared with 39 (45.3%) residents; however, this difference did not reach statistical significance (p=0.054). Diagnostic correctness was significantly associated with years of clinical experience (p=0.022), as physicians with more than 10 years of experience achieved the highest rate of complete correctness at nine (90.0%), followed by those with 5-10 years at 17 (77.3%), whereas only 37 (44.0%) of those with one to five years achieved full correctness. A significant gender difference was observed (p=0.013), with 42 (66.7%) female physicians correctly diagnosing all three cases compared with 21 (39.6%) male physicians. Self-rated orthopedic knowledge demonstrated a strong positive gradient with diagnostic accuracy (p<0.001), as full correctness increased from two (15.4%) among those rating their knowledge as very poor to 15 (83.3%) among those rating it as good and one (100.0%) among those rating it as very good. Awareness of referral criteria was also strongly associated with diagnostic accuracy (p<0.001), with 48 (72.7%) of aware physicians achieving correct diagnosis in all three cases compared with only 15 (30.0%) among those who were not aware.

**Table 4:** Correctness of diagnosis across qualification, experience, gender, self-rated knowledge, and referral-criteria awareness † p value for Chi-square test; *p<0.05 considered significant.

Physician Characteristic	Category	0 Correct Dx (None)	1 Correct Dx	2 Correct Dx	3 Correct Dx (All)	Pearson Chi-Square (X^2^)	p value†
Qualification/Position	Consultant	0 (0.0%)	0 (0.0%)	3 (25.0%)	9 (75.0%)	12.405	0.054
	Specialist	0 (0.0%)	0 (0.0%)	3 (16.7%)	15 (83.3%)		
	Resident	1 (1.2%)	14 (16.3%)	32 (37.2%)	39 (45.3%)		
Years of Experience	1-5 years	1 (1.2%)	14 (16.7%)	32 (38.1%)	37 (44.0%)	14.812	0.022*
	5-10 years	0 (0.0%)	0 (0.0%)	5 (22.7%)	17 (77.3%)		
	>10 years	0 (0.0%)	0 (0.0%)	1 (10.0%)	9 (90.0%)		
Gender	Female	1 (1.6%)	4 (6.3%)	16 (25.4%)	42 (66.7%)	10.737	0.013*
	Male	0 (0.0%)	10 (18.9%)	22 (41.5%)	21 (39.6%)		
Self-Rated Ortho Knowledge	1 (Very poor)	1 (7.7%)	7 (53.8%)	3 (23.1%)	2 (15.4%)	55.712	<0.001*
	2 (Poor)	0 (0.0%)	5 (18.5%)	16 (59.3%)	6 (22.2%)		
	3 (Fair)	0 (0.0%)	2 (3.5%)	16 (28.1%)	39 (68.4%)		
	4 (Good)	0 (0.0%)	0 (0.0%)	3 (16.7%)	15 (83.3%)		
	5 (Very good)	0 (0.0%)	0 (0.0%)	0 (0.0%)	1 (100.0%)		
Referral-Criteria Awareness	No	1 (2.0%)	14 (28.0%)	20 (40.0%)	15 (30.0%)	30.769	<0.001*
	Yes	0 (0.0%)	0 (0.0%)	18 (27.3%)	48 (72.7%)		

Attitude toward appropriate orthopedic management, measured using a composite score (0-5), differed significantly across several physician characteristics (Table 5). A highly significant difference was noted by qualification level (p<0.001), with consultants (4.17 ± 1.19) and specialists (4.06 ± 1.00) demonstrating substantially more appropriate management attitudes compared to residents (2.28 ± 1.34). Similarly, post-internship clinical experience showed strong significance (p<0.001), wherein physicians with 5-10 years (3.27 ± 1.58) and >10 years of experience (4.40 ± 0.70) scored higher than those with one to five years of experience (2.42 ± 1.39). Female physicians exhibited significantly more favorable management attitudes than male physicians (p=0.004), reflected by higher mean scores (3.11 ± 1.54 vs. 2.32 ± 1.36). Self-perceived orthopedic knowledge had a strong positive gradient with attitude score and was statistically highly significant (p<0.001), with physicians rating their knowledge as “Good” or “Very Good” achieving the highest appropriate management scores, including perfect appropriateness among the single physician self-rating “Very Good” (5.00 ± 0.00). Importantly, awareness of established referral criteria was also strongly associated with appropriate management attitudes (p<0.001), with aware physicians scoring notably higher (3.61 ± 1.25) compared to those not aware (2.42 ± 1.39).

**Table 5:** Attitude/management score (0-5) across physician characteristics † p values from group comparison tests (t=Students ‘t’ test;  F=Analysis of Variance); p<0.05 considered significant.

Physician Characteristic		Category	Mean Score ± SD	Minimum	Maximum	Test statistics (t/F)	p value†
Qualification/Position		Consultant	4.17 ± 1.19	2.00	5.00	22.439 (F)	<0.001
		Specialist	4.06 ± 1.00	2.00	5.00		
		Resident	2.28 ± 1.34	0.00	5.00		
Years of Experience		1-5 years	2.42 ± 1.39	0.00	5.00	11.075 (F)	<0.00
		5-10 years	3.27 ± 1.58	0.00	5.00		
		>10 years	4.40 ± 0.70	3.00	5.00		
Gender		Female	3.11 ± 1.54	0.00	5.00	2.912 (t)	0.004
		Male	2.32 ± 1.36	0.00	5.00		
Self-Rated Ortho Knowledge		Very poor	1.38 ± 1.26	0.00	4.00	8.485 (F)	<0.001
		Poor	2.07 ± 1.21	0.00	5.00		
		Fair	3.07 ± 1.44	0.00	5.00		
		Good	3.61 ± 1.24	2.00	5.00		
		Very good (1)	5.00 ± 0.00	5.00	5.00		
Referral Awareness		Aware	3.61 ± 1.25	0.00	5.00	-4.205 (t)	<0.001
		Not Aware	2.42 ± 1.39	0.00	5.00		

## Discussion

In our study, we assessed the knowledge and attitudes of family physicians in the Eastern Province toward the most common orthopedic surgery cases. Accordingly, the discussion is organized into three domains: inaccurate diagnosis, inappropriate management, and reasons for referral.

Inaccurate diagnosis

Primary healthcare physicians frequently refer patients with osteoarthritis to orthopedic clinics. While they play a crucial role in diagnosing and managing osteoarthritis, many still lack adequate knowledge and confidence in managing and diagnosing these cases. This was demonstrated in a study conducted in Saudi Arabia, where only 49.7% of participants answered osteoarthritis-related questions correctly. In contrast, our study showed that approximately 108 (93%) physicians could accurately diagnose osteoarthritis. In addition to osteoarthritis, adhesive capsulitis is another orthopedic condition commonly encountered by primary healthcare physicians. As it is a very important condition, a study conducted in the province of Quebec, Canada, reported that 99% of physiotherapists and 95% of family physicians could correctly identify adhesive capsulitis. Comparatively, 95 (81%) of our respondents reached the correct diagnosis. Regarding developmental dysplasia of the hip (DDH), a study to assess the knowledge, attitude, and practice of primary care physicians in a tertiary hospital in Riyadh, Saudi Arabia, reported that 69.2% of physicians possessed sufficient knowledge of the condition. Similarly, our study found that 76 (65.5%) were correctly diagnosed with DDH. Misdiagnosis of DDH in pediatric patients is concerning, as it can significantly affect quality of life and lead to complications such as limb-length discrepancy, waddling gait, chronic low back pain, and early-onset osteoarthritis [7,10-12].

Inappropriate management

Primary healthcare physicians play a key role in patient satisfaction and the efficiency of healthcare systems. Deciding whether to manage a condition at the primary care level or refer the patient to a specialist is often complex and may either improve outcomes or contribute to unnecessary healthcare burden and prolonged waiting times. Unnecessary referrals also occupy appointment slots that could otherwise be allocated to patients who genuinely require more specialized care.

In our study, we evaluated how primary healthcare physicians managed common orthopedic cases and whether they appropriately treated or referred patients in need. The questionnaire focused on osteoarthritis, DDH, adhesive capsulitis, scoliosis, and disc prolapse-conditions frequently encountered in primary care. Regarding osteoarthritis, Homoud AA showed that only 36.4% of physicians continued treatment at the primary care level [10]. Conversely, in our study, 83 (71.6%) recommended appropriate conservative management (lifestyle modification, analgesics, and physiotherapy referral), while only four (3.4%) opted for direct referral to orthopedic services [10].

For DDH, 71 (61.2%) of physicians in our study appropriately recommended ultrasound and orthopedic referral. However, 31 (26.7%) preferred re-evaluating the patient after two months without imaging or referral. In contrast with a study in Riyadh, Saudi Arabia, where 90.8% of physicians indicated they would only refer after positive imaging findings, a nearly 30% discrepancy from our results. Similarly, Théroux et al. demonstrated that only 24% of family physicians felt comfortable managing DDH independently [12]. Moreover, regarding scoliosis, about 59 (50.9%) of respondents indicated they would reassure patients and recommend exercise, whereas only 47 (40.5%) selected orthopedic referral for a Cobb angle >48°, which is the appropriate action. In terms of adhesive capsulitis management, Lowry et al. found that approximately 76% of family physicians would refer patients to physiotherapy and prescribe NSAIDs [10]. Our findings were comparable, with 81 (69.8%) selecting this approach. Regarding acute low back pain, a study reported that only 10.6% of primary care physicians referred all such cases to higher-level care. In our study, 74 (63.8%) advised bed rest, which is not recommended, while only 38 (32.8%) selected orthopedic referral during a second visit if pain was severe or limited function [7,11-14].

Reasons for referral

Most referrals to orthopedic surgery are prompted by knee pain, back pain, and, to a lesser extent, shoulder pain and hallux valgus. However, recent literature suggests that many of these referrals are inappropriate. Notably, 74.5% of referrals to pediatric orthopedic clinics were inconsistent with AAP guidelines. Moreover, many conditions seen in pediatric orthopedic clinics represent normal variants or minor MSK issues that can be adequately managed in primary care settings. This was reflected in a study conducted among pediatricians and family physicians, where 87.3% of pediatricians and general practitioners lacked adequate knowledge of common pediatric orthopedic conditions. In contrast, our results showed that 31 (26.7%) of PCPs misdiagnosed DDH as physiological bowing, and 14 (12.1%) advised double-diapering, while 31 (26.7%) opted to delay referral for two months. In addition, the previous two studies have shown that patients’ insistence is a common factor influencing unnecessary referrals. In contrast, our study found that patients’ request was the least common reason for referral. Moreover, other referrals primarily resulted from diagnostic uncertainty. Importantly, consistent with Robarts et al. [15], patient gender did not influence referral decisions [2-4,6,16].

As with any study, this research has certain limitations. First, it was conducted in a single region, the Eastern Province of Saudi Arabia, which may limit the generalizability of the findings to the rest of the country. Additionally, the study did not specify whether participants had received formal teaching sessions or completed clinical rotations in orthopaedic surgery during their training. Furthermore, most participants were residents, which may further limit the generalizability of the results. Finally, formal pilot testing and psychometric reliability analyses of the questionnaire were not conducted prior to data collection; this is acknowledged as a limitation of the study. However, the questionnaire was reviewed and validated by three consultants.

## Conclusions

Family physicians in the Eastern Province of Saudi Arabia demonstrate a fair level of knowledge regarding common orthopedic conditions. However, notable gaps persist that may influence patient care and referral practices. Targeted educational initiatives are recommended to bridge these knowledge and skill gaps and strengthen the competencies of family physicians. Furthermore, establishing clear and structured orthopedic referral guidelines is crucial to prevent overwhelming orthopedic clinics with unnecessary referrals, long waiting lists, and prolonged follow-up appointments. Promoting the use of standardized referral pathways will support appropriate patient triage and enhance the overall efficiency of orthopedic services.

## Appendices

The questionnaire used for data collection is presented in Table 7, which presents the demographic characteristics of the participants, and in Table 8, which presents the questions used to assess the participants' orthopedic knowledge.

**Table 6:** Participants' demographics The demographics questionnaire was created and edited by the authors Dalal Albaiji, Manar Alossaif, and Ammar Alomran. Furthermore, it was revised and approved by three consultants.

Gender	Female
	Male
Qualification/ position	Consultant
	Specialist
	Resident
If resident, year of residency (n=86)	R1
	R2
	R3
If consultant (any fellowship) n=12	No
	Yes
Years of Experience (After Internship)	1-5 Years
	5-10 Years
	More Than 10 Years
Training/Employment Center	Ministry of health
	IAU Family and community center
	Armed Forces Hospital - Dhahran
	JHAH
	KFMMC - Dhahran
	Royal Commission Hospital- Jubail
How much do you rate your knowledge in orthopedic-related conditions on a scale (1-5)	1 (Very Poor)
	2 (Poor)
	3 (Fair)
	4 (Good)
	5 (Very Good)
Are you aware if there are any referral criteria related to any orthopedic-related condition	No
	Yes

**Table 7:** A questionnaire-based assessment of orthopedic knowledge in PCPs The questionnaire was created and edited by the authors Dalal Albaiji, Manar Alossaif, and Ammar Alomran. Furthermore, it was revised and approved by three consultants.

Case Scenario	Domain	Response Option
A 64-year-old male presented to the clinic complaining of pain in his knees that started 1 year ago. Upon further questioning, the pain is limited to the anterior and medial sides bilaterally. It is also difficult to bend his knees when he is sitting on the floor, and climbing the stairs makes the pain worse. On local examination: there were no scars, skin rashes or erythema. However, there was varus deformity bilaterally and limited range of motion with crepitus on passive movement.	Knowledge	A. Rheumatoid arthritis
		B. Septic knee
		C. Osteoarthritis*
		D. Patellofemoral pain syndrome
	Attitude	A. Refer as patient’s request
		B. Lifestyle modification + NSAIDs
		C. Refer to physiotherapy
		D. Both B & C*
A 2-month-old baby girl came to the vaccination clinic, while you were evaluating her you noticed leg length discrepancy, you did a hip examination and you noticed limitations in hip abduction.	Knowledge	A. Physiological bowing
		B. SCFE
		C. Developmental Dysplasia of Hip*
		D. Proximal femoral focal deficiency
	Attitude	A. Double diaper advice
		B. Do US + refer to Orthopedic surgery*
		C. Follow-up 2 months + re-evaluate
		D. Reassurance only
An 18-year-old girl presented to your clinic after her mother noticed a shoulder imbalance. Upon further questioning there were no respiratory symptoms, no walking issue and no back pain. On examination: there were no skin dimples, no rash, no scars, or hairy patches. You performed Adam’s forward bending test and there was noticeable asymmetry. You ordered X-Ray and the image confirms the presence of bend to the right and cobb’s angle >48 degrees.	Attitude	A. Reassure + stretching
		B. Refer to Orthopedic surgery*
		C. Order Vit D, Ca, PO₄, PTH
		D. Encourage bracing
A 50-year-old woman with diabetes presents with a 2-month history of insidious onset right shoulder pain. She denies a history of shoulder trauma. She has no history of neck pain, arm/hand weakness, numbness or paresthesia. The pain is felt at extremes of range of motion and has difficulty sleeping on the affected side. She has noticed increasing difficulty with activities of daily living, including brushing her hair, as well as putting on or taking off her clothes. Her examination shows a marked decrease in both active and passive range of motion of the right shoulder.	Knowledge	A. Rotator cuff tear
		B. Frozen shoulder*
		C. Shoulder impingement
		D. Glenohumeral OA
	Attitude	A. Physiotherapy alone
		B. Ortho referral on 1st request
		C. NSAIDs alone
		D. Both A & C*
A 26-year-old female presents with acute low back pain. She says it started a week ago after she lifted a sofa when helping a friend move. The patient's medical history is otherwise negative. The patient says the pain is limited to the lower back. The physical examination is normal, including the neurologic examination.	Attitude	A. Bed rest
		B. Twisting/bending only
		C. Immediate ortho referral
		D. Ortho referral 2nd visit if severe or limits function*
Generally in your practice, what would make you refer a case to orthopedic surgery? (you can choose multiple options)	Attitude	A. Patients’ request
		B. Unsure of the diagnosis
		C. The need for further investigations (labrotary invistigations and imaging)
		D. Not responding to initial management
		E. No avaliable physiothyrapists in the center
